# Determining the Mental Health Literacy Level of University Students and Examining Their Attitudes Towards Seeking Psychological Help

**DOI:** 10.1111/inm.13506

**Published:** 2025-01-21

**Authors:** Nevra Didem Kılınç, Gülcan Kendirkıran

**Affiliations:** ^1^ Koç University Institute of Health Sciences İstanbul Turkey; ^2^ Istanbul Arel University Faculty of Health Sciences Department of Nursing İstanbul Turkey; ^3^ Department of Nursing Haliç University Faculty of Health Sciences İstanbul Turkey

**Keywords:** health literacy, help‐seeking behaviour, mental health, psychological wellbeing, university students

## Abstract

This descriptive study examined the mental health literacy levels and attitudes towards seeking psychological help among university students. The study was conducted with 317 students from a university in Türkiye. Data were collected via an online platform using a Personal Information Form, the Mental Health Literacy Scale (MHLS) and the Attitudes Toward Seeking Professional Psychological Help Scale‐Short Form (ATSPPHS‐SF). Participants, with an average age of 20 ± 1, were predominantly female (89%). Most had previously sought psychological support (75.1%) and believed the psychology information obtained online was accurate (61.2%). Significant differences were found between residential location (*p* = 0.034) and maternal education level (*p* = 0.017) with the Knowledge Subscale of MHLS, and between history of seeking psychological support (*p* = 0.010) with the Resource Subscale of MHLS. Additionally, there was a significant difference between seeking psychological support history and mean ATSPPHS‐SF scores (*p* = 0.060). The scores on MHLS (13.15 ± 3.38) and ATSPPHS‐SF (18.38 ± 3.77) indicated moderate levels, with a positive and significant relationship between the scales' total scores (*p* = 0.000). The study concluded that mental health literacy is crucial for increasing the rate of seeking psychological help. Addressing the lack of knowledge regarding mental health is essential for early diagnosis, prevention and maintenance of mental health. It is recommended incorporating mental health literacy content into university curricula, initiating efforts to provide information about psychological help resources and engaging psychiatric nurses in educational roles on campus through multidisciplinary planning.

## 1 Introduction

Mental health literacy (MHL) is a critical component of overall health literacy, defined as the ‘knowledge and beliefs about mental disorders that aid in their recognition, management, and prevention’ (Jorm et al. 1997). It encompasses the ability to recognise symptoms of mental disorders, understand the available treatment options, and know where to seek help. MHL has the potential to positively influence both individual and community well‐being by promoting actions for the prevention, early intervention and treatment of mental health issues (Özel and Duzcu 2018). Psychological help‐seeking behaviour, on the other hand, involves individuals seeking resources to cope with events that pose psychological threats (Fisher and Turner 1970).

The university period, which coincides with late adolescence, is a phase where young adults are exposed to numerous new experiences. This period is significant for acquiring social, academic, professional and personal skills, but it is also a time when many face mental health challenges (Gürsoy and Gizir 2018). University students often experience higher levels of psychological distress compared to their non‐university peers due to academic pressures, the need to establish new social networks, financial worries and the task of maintaining their daily routines (Rababah et al. 2019). Mental health knowledge plays a crucial role in predicting help‐seeking behaviours and the likelihood of disclosing mental health issues to family and friends, thereby underscoring the importance of MHL (Henderson, Evans‐Lacko, and Thornicroft 2013).

A deficiency in MHL adversely impacts young adults' development and increases the risk of psychiatric relapses (Nobre et al. 2021). Factors influencing attitudes towards seeking psychological help include gender, age, personal or familial experiences with psychological services, economic status and perceived levels of social support (Aydın 2017). Some characteristics, such as gender, age, exercise and dietary behaviours, require more attention as these factors are predictors of a positive mental health (Nogueira et al. 2022). Studies have shown that individuals with prior experience of psychological help tend to have more favourable attitudes towards seeking such help (Meydan and Lüleci 2013). Friends and family are often preferred sources of support over professionals, but those who opt for professional assistance usually exhibit positive attitudes towards help‐seeking, driven by social encouragement and established trust with mental health experts (Gulliver, Griffiths, and Christensen 2010).

## 2 Background

The prevalence of mental health disorders among university students is a growing concern. Anxiety disorders are among the most common psychiatric problems in this population, with approximately 11.9% of students affected (González‐Valero et al. 2019). Additionally, depression is prevalent, with an incidence rate of 7%–9% among students, often linked to decreased academic performance, increased substance abuse and a higher risk of suicidal behaviour (Eisenberg, Hunt, and Speer 2013). Suicide, the third leading cause of death among young individuals, is a critical issue among university students (Gselamu and Ha 2020). Moreover, attention deficit hyperactivity disorder (ADHD), characterised by inattention, hyperactivity and impulsivity, poses significant academic, social and psychiatric challenges for students (Sedgwick 2017). ADHD often begins in childhood and continues into adulthood, negatively impacting various aspects of life (Mohammadi et al. 2021). Eating disorders, such as anorexia nervosa and bulimia nervosa, also commonly affect university students. These disorders typically begin during adolescence and can escalate during early adulthood, leading to severe health complications (Manning and Greenfield 2022). Factors such as unhealthy eating habits, lack of sleep, stress and peer pressure can adversely affect students' health (Rababah et al. 2019).

Awareness and knowledge about mental health issues and the benefits of seeking help are crucial. Individuals who are aware of mental health problems and the available resources are more likely to seek help when needed (Siddique et al. 2022). Personal beliefs and attitudes, including perceptions about the effectiveness of treatment and self‐stigma, also significantly influence help‐seeking behaviours (Mahsoon et al. 2020).

Access to mental health care is another critical factor. Students who lack access to mental health services or face barriers in obtaining care are less likely to seek help (McCarty et al. 2006). Enhancing mental health literacy and addressing these barriers can encourage more students to seek the help they need.

Research indicates that mental health knowledge and attitudes towards seeking help are influenced by several demographic and psychosocial factors. Gender differences play a significant role, with female students generally displaying higher levels of mental health literacy and more favourable attitudes towards seeking psychological help compared to their male counterparts (Singh et al. 2022). Cultural factors also impact attitudes towards mental health and help‐seeking behaviours. In some cultures, mental health issues may be stigmatised or viewed as a sign of personal weakness, deterring individuals from seeking help (Zhou et al. 2022).

Universities play a crucial role in promoting mental health literacy and providing support to students. Educational institutions are well‐positioned to implement programmes that enhance students' knowledge about mental health, reduce stigma, and encourage help‐seeking behaviours. For example, psychoeducation programmes can provide valuable information about mental health disorders, their symptoms and the importance of early intervention (Alhadidi et al. 2020). Cognitive‐behavioural therapy (CBT) has been shown to be effective in treating anxiety and depression among university students by helping them identify and change negative thought patterns and behaviours (Oar, Johnco, and Ollendick 2017).

Group therapy is another effective approach, offering social support and improving coping skills while addressing various mental health issues such as anxiety, depression and substance abuse (Hartley, Redmond, and Berry 2022). Additionally, pharmacological interventions play a significant role in managing mental health disorders. Educating students and their families about medication, monitoring side effects and encouraging adherence to treatment are vital components of psychiatric nursing (Earle 2016).

Mindfulness‐based approaches have also proven beneficial in reducing stress, anxiety and depressive symptoms among students. These approaches help individuals develop greater self‐awareness and improve overall well‐being (Lin, Chadi, and Shrier 2019). Health professionals' attitudes and communication skills significantly impact the quality of care provided to young adults. Supportive and understanding interactions with health care providers can encourage students to seek and continue receiving mental health care (Alkhawaldeh 2017).

Improving mental health literacy among university students is crucial for early diagnosis and intervention. Students who are knowledgeable about mental health issues and understand the importance of seeking help are more likely to take appropriate action when they or their peers experience mental health problems (Reavley, McCann, and Jorm 2012). Effective mental health literacy programmes can enhance students' decision‐making abilities and prevent the development of mental health disorders (Ahmad et al. 2022).

In summary, mental health literacy is a vital aspect of health education that can significantly impact the well‐being of university students. By recognising the symptoms of mental disorders, understanding treatment options and knowing where to seek help, students can improve their mental health outcomes. Universities have a responsibility to provide education and support that enhances mental health literacy and encourages help‐seeking behaviours, thereby fostering a healthier student population. In this descriptive study, it was aimed to examine the mental health literacy levels and attitudes towards seeking psychological help among university students.

### 2.1 Participants

The study involved 317 students from four departments of the Faculty of Health Sciences at a university in Istanbul, Türkiye. Initially, the study targeted a population of 1658 students. The criteria for inclusion were being a Turkish citizen and being actively enrolled in the university during the data collection period. The final sample size was determined with an *α* value of 0.05, a *t* value of 1.96 and a *p* value of 0.50 (Cochran 1977).

### 2.2 Instruments

Data were collected using three primary instruments:

1. Personal Information Form: Developed by the researchers after reviewing relevant literature (Koçyiğit and Pamukçu 2018; Kaya, Yücel, and Demirtaş‐Baysal 2019), this form comprised 13 questions. It gathered sociodemographic information and details about the participants' psychological/psychiatric support history.
2. Mental Health Literacy Scale (MHLS): Originally created by Jung, von Sternberg, and Davis (2016) and translated into Turkish with validation by Göktaş et al. (2019). The scale measures mental health literacy across three sub‐dimensions: Knowledge (12 items), Beliefs (10 items) and Resources (4 items). Responses are rated on a Likert scale ranging from ‘strongly agree’ to ‘strongly disagree’ and ‘yes’ or ‘no’ for the resource dimension. The total possible score ranges from 0 to 26, with higher scores indicating greater mental health literacy. The Cronbach's alpha for this study was 0.73.
3. Attitudes Toward Seeking Professional Psychological Help Scale‐Short Form (ATSPPHS‐SF): Developed by Fischer and Farina (1995) and adapted to Turkish by Topkaya (2012). This 10‐item scale assesses attitudes towards seeking professional psychological help, using a 4‐point Likert scale from ‘strongly agree’ to ‘strongly disagree’. The total possible score ranges from 0 to 27, with higher scores reflecting more positive attitudes. Items 2, 8, 9 and 10 are reverse‐scored. The Cronbach's alpha for this scale in the current study was 0.66.

### 2.3 Procedure

Data collection was conducted between 28 March and 26 April 2023, following ethical approval from the Haliç University Non‐Invasive Clinical Research Ethics Committee (Approval No: 2022/26) and institutional permission (Approval No: 74). The researchers uploaded the survey instruments to an online platform and shared the access link with students. Participation was voluntary, and students were informed about the study's objectives, procedures and confidentiality measures through an informed consent form included at the beginning of the online survey. Each participant could submit their responses only once after accepting the informed consent form online, to ensure data integrity.

### 2.4 Data Analysis

The collected data were analysed using SPSS version 25.0. The analysis included both descriptive and inferential statistics. Descriptive statistics (frequency, percentage, mean and standard deviation) were used to summarise the participants' sociodemographic characteristics and their scores on the MHLS and ATSPPHS‐SF.

The normality of the data distributions was assessed using the Kolmogorov–Smirnov test. Due to non‐normal distributions, non‐parametric tests were employed for further analysis. The Mann–Whitney *U*‐test and Kruskal–Wallis test were used to examine differences in mental health literacy and attitudes towards seeking professional psychological help across various sociodemographic groups. Pearson correlation analysis was conducted to explore the relationship between mental health literacy and attitudes towards seeking professional psychological help. Statistical significance was set at *p* < 0.05 for all analyses.

## 3 Results

### 3.1 Demographic Characteristics, MHLS and ATSPPHS

In the study, the average age of participants was 20 ± 1 years. Among the participants, 89% were female, 61.2% were nursing students and 60.3% were first‐year students. It was found that 75.7% of participants lived with their families and 75.1% had not previously received psychological support. Among those who had received psychological support, 44.4% received it from a psychological counsellor. 19.6% of participants obtained information about psychology through Google searches and 61.2% believed the information accessed online about psychology/psychiatry was accurate (Table 1). The mean scores on the MHLS subscales were found to be 7.94 ± 2.08 for Knowledge‐Oriented MHLS, 2.51 ± 2.1 for Belief‐Oriented MHLS, 2.69 ± 1.13 for Resource‐Oriented MHLS, with a total MHLS score of 13.15 ± 3.38. The mean score on the ATSPPHS was 18.38 ± 3.77 (Table S1).

**TABLE 1:** Descriptive characteristics of participants.

Variables (*n* = 317)	Mean ± SD	Min	Max
Age	20 ± 1	18	27

	*n*	%
Gender		
Female	282	89
Male	35	11
Department		
Nutrition and dietetics	66	20.8
Midwifery	46	14.5
Physiotherapy and rehabilitation	11	3.5
Nursing	194	61.2
Grade		
1st grade	191	60.3
2nd grade	77	24.3
3rd grade	22	6.9
4th grade	27	8.5
Employment status		
Working	55	17.4
Not working	262	82.6
Living situation		
Living alone	14	4.4
Living in a dormitory	47	14.8
Living with family	240	75.7
Living with roommate/friends	16	5
Mother's education level		
Literate	18	5.7
Primary school	169	53.3
High school	99	31.2
Associate degree	6	1.9
Undergraduate	23	7.3
Postgraduate	2	0.6
Father's education level		
Literate	6	1.9
Primary school	160	50.5
High school	88	27.8
Associate degree	14	4.4
Undergraduate	40	12.6
Postgraduate	9	2.8
Took psychology course in undergraduate education		
Yes	157	49.5
No	160	50.5
Previously received psychological support		
Yes	79	24.9
No	238	75.1
Person providing psychological support		
Psychologist	0	0
Psychiatrist	24	29.6
Psychological counsellor	36	44.4
Multiple experts	6	7.4
Sources of psychology information		
Instagram	167	17.3
Twitter	62	6.4
Facebook	7	0.7
WhatsApp groups	17	1.8
From doctors' pages	109	11.3
From hospital pages	71	7.4
I search on Google	189	19.6
From books	152	15.8
From people around me	110	11.4
From health care professionals in clinical practice	60	6.2
Other	20	2.1
Accuracy of psychology information on the internet		
True	194	61.2
False	123	38.8

### 3.2 Relationship Between MHLS and ATSPPHS

A statistically significant, positive but weak relationship was found between age and the Knowledge‐Oriented MHLS subscale scores (*p* = 0.00). No statistically significant relationship was found between age and the Belief‐Oriented MHLS subscale, Resource‐Oriented MHLS subscale, total MHLS score or ATSPPHS total score (*p* > 0.05) (Table S2). A statistically significant, weak, positive relationship was found between the total ATSPPHS score (*p* = 0.00) and the Knowledge‐Oriented MHLS subscale (*p* = 0.00), Belief‐Oriented MHLS subscale (*p* < 0.01) and total MHLS score (*p* = 0.00) (Table 2).

**TABLE 2:** Relationship between participants' MHLS total and sub‐dimension and ATSPHS total score averages.

Scale (*n* = 317)	ATSPHS—Total	
MHLS—*Knowledge‐oriented*	*r*	**0.217**
	*p*	**0.000**
MHLS—*Belief‐oriented*	*r*	0.188
	*p*	**0.001**
MHLS—*Resource‐oriented*	*r*	0.077
	*p*	0.174
MHLS—Total	*r*	0.316
	*p*	**0.000**

Abbreviations: ATSPPHS, attention toward seeking professional psychological help scale; MHLS, mental health literacy scale; *r*, Pearson correlation; bold vales, MHLS Knowledge‐oriented = *p* 0.000, MHLS Belief‐oriented = *p* 0.001, MHLS Total = *p* 0.000 .

### 3.3 Comparison of MHLS With Demographic Characteristics

The Knowledge‐Oriented MHLS subscale scores varied significantly (*p* = 0.00) by the participants' living situation and mother's education level. Post‐hoc analysis revealed that those living in dormitories scored higher than those living alone, and those whose mothers had postgraduate education scored higher than those whose mothers had undergraduate, high school or associate degrees. No significant differences were found in the Knowledge‐Oriented MHLS subscale scores based on gender, employment status, taking a psychiatry or psychology course, previous psychological support, belief in the accuracy of online psychology/psychiatry information, department, year of study, father's education level or type of psychological support received (*p* > 0.05) (Table S3). No significant differences were found in the Belief‐Oriented MHLS subscale scores based on gender, employment status, taking a psychiatry or psychology course, previous psychological support, belief in the accuracy of online psychology/psychiatry information, department, year of study, living situation, mother's education level, father's education level or type of psychological support received (*p* > 0.05) (Table S4). The Resource‐Oriented MHLS subscale scores were significantly higher among those who had previously received psychological support (*p* = 0.01). No significant differences were found based on gender, employment status, taking a psychiatry or psychology course, belief in the accuracy of online psychology/psychiatry information, department, year of study, living situation, mother's education level, father's education level or type of psychological support received (*p* > 0.05) (Table S5). In the study, no statistically significant differences were found between the participants' total MHL score averages and the variables of gender, employment status, taking a course related to psychiatry or psychology during undergraduate education, previous psychological support, believing that the psychology/psychiatry information accessed online is accurate, department, year of study, living situation, mother's education level, father's education level and the person from whom psychological support is received (*p* > 0.05) (Table S6).

### 3.4 Comparison of ATSPPHS With Demographic Characteristics

The total ATSPPHS scores were significantly higher among those who had previously received psychological support (*p* = 0.00). No significant differences were found in the total ATSPPHS scores based on gender, employment status, taking a psychiatry or psychology course, belief in the accuracy of online psychology/psychiatry information, department, year of study, living situation, mother's education level, father's education level or type of psychological support received (*p* > 0.05) (Table S7).

## 4 Discussion

University life is a period characterised by numerous new experiences for young adults, who face a variety of social, academic, professional and personal challenges. These challenges often prompt students to seek help (Gürsoy and Gizir 2018). During this period, university students are considered a risk group for experiencing mental health issues (O'Neill et al. 2018). High levels of mental health literacy (MHL) can have positive effects on recognising actions for prevention, early intervention and treatment of psychological disorders (Özel and Duzcu 2018). Thus, MHL significantly impacts whether students with psychological problems receive the necessary help.

### 4.1 MHLS and ATSPPHS

In this study, the average score obtained from the MHLS by participants was 13.15 ± 3.38. This finding is consistent with previous research. For example, Göktaş et al. (2019) conducted a study with 215 students at a university in Türkiye and reported an average MHLS score of 12.06 ± 3.80. Another study by Kantaş Yılmaz and Ünkür (2023) involving 2791 health sciences students in Istanbul found an average MHLS score of 14.53 ± 3.31. Additionally, a study by Tunç Karaman, Altun, and Basat (2022) conducted with 327 individuals aged 18–65 at a family medicine clinic reported an average MHLS score of 14.05 ± 3.49. These results indicate that the MHLS scores in this study are similar to those in other studies in the literature. It suggests that university students may have a lower average level of MHL than the general population.

The average score obtained by participants from the ATSPPHS was 18.38 ± 3.77. In comparison, a study by Rayan, Baker, and Fawaz (2020) conducted with 519 students at eight universities in Jordan reported an average ATSPPHS score of 15.7 ± 4. Another study by Altundağ, Kılıç, and Biçer (2021) involving 884 students at a university in Türkiye found an average ATSPPHS score of 27.93 ± 4.34. The ATSPPHS score in this study is similar to the findings of Rayan, Baker, and Fawaz (2020) but lower than that reported by Altundağ, Kılıç, and Biçer (2021). The difference in scores may be attributed to the inclusion of students from different faculties in the sample group.

This study found a weak but positive correlation between participants' attitudes towards seeking psychological help and their knowledge‐oriented, belief‐oriented and overall MHL levels. This finding aligns with other studies. For instance, Kantaş Yılmaz and Ünkür (2023) studied health sciences students and found a similar relationship between attitudes towards seeking psychological help and MHL. Additionally, Almanasef (2021) conducted a study with 1300 university students in Saudi Arabia and reported a positive and weak relationship between help‐seeking behaviour and MHL. Gorczynski et al. (2017) conducted a study with 379 participants at a university in England and also found that MHL positively correlated with help‐seeking behaviour. These findings suggest that as MHL increases, so do positive attitudes towards seeking psychological help. Individuals with higher knowledge and awareness of mental health are more likely to seek help when needed. This is possibly because they are more aware of the benefits of early intervention and treatment, reducing the stigma associated with seeking help.

### 4.2 MHLS With Demographic Characteristics

In this study, knowledge‐oriented MHL was found to be higher among students living in dormitories compared to those living alone. Pehlivan, Tokur Kesgi̇n, and Uymaz (2020) conducted a study with 417 university students in Türkiye and found no significant difference in MHL levels based on living arrangements. On the other hand, Abonassir et al. (2021) found that students living with their families had higher MHL levels. These mixed results suggest that the relationship between living arrangements and MHL requires further investigation. It could be hypothesised that communal living arrangements like dormitories may facilitate the sharing of mental health information and support among peers, thereby increasing MHL. Future studies exploring this relationship can contribute valuable insights to the literature.

The study also found that knowledge‐oriented MHL was higher among students whose mothers had postgraduate education compared to those whose mothers had undergraduate, high school or associate degrees. This finding is consistent with Abonassir et al. (2021), who reported that participants with highly educated mothers had higher MHL. The increase in education level is likely associated with greater engagement in information‐seeking and awareness, contributing to higher MHL. Educated parents might provide a more supportive environment for discussing mental health issues, encouraging their children to seek help when needed.

Additionally, this study found that resource‐oriented MHL subscale scores were higher among students who had previously received psychological support. Gorczynski et al. (2017) reported similar findings, indicating that students diagnosed with psychiatric disorders had higher MHL. Pehlivan, Tokur Kesgi̇n, and Uymaz (2020) found no significant difference in MHL between those who had and those who had not received psychiatric help. It can be inferred that knowledge about available resources can encourage help‐seeking behaviour when mental health is at risk. Prior experience with psychological support may also increase familiarity with mental health services, reducing barriers to seeking help in the future.

No significant differences were found between knowledge‐oriented, belief‐oriented, resource‐oriented and overall MHL levels and gender. Pehlivan, Tokur Kesgi̇n, and Uymaz (2020) also found no significant gender differences in MHL. However, Kantaş Yılmaz and Ünkür (2023) found higher knowledge‐oriented, belief‐oriented and overall MHL levels among female students. Similarly, Almanasef (2021) reported higher MHL levels among female students compared to males. Gorczynski et al. (2017) found that female students had higher MHL levels. The difference may be attributed to the societal tendency for women to express their emotions more openly and thus be more willing to engage in mental health‐related activities. Women might be more proactive in seeking information and help for mental health issues, contributing to higher MHL.

### 4.3 ATSPPHS With Demographic Characteristics

This study found that students who had previously received psychological support had more positive attitudes towards seeking psychological help. This finding is supported by Seyfi et al. (2013) and Kantaş Yılmaz and Ünkür (2023), who found that students with no prior psychological support had higher levels of negative attitudes towards seeking help. Previous experience with psychological support likely provides knowledge and experience of the process, positively influencing attitudes towards seeking help. Students who have previously sought psychological support might be more familiar with the benefits and processes of receiving help, which can reduce fear and stigma associated with seeking psychological services.

Additionally, no significant differences were found between attitudes towards seeking psychological help and employment status. It can be hypothesised that students balancing academic responsibilities and work might have varied attitudes towards seeking help. Employment might add to the stress levels of students, influencing their attitudes towards seeking psychological help. However, further research is needed to explore this relationship comprehensively. Understanding the impact of employment on students' attitudes towards seeking help can inform interventions to support working students better.

No significant differences were found between attitudes towards seeking psychological help and gender. Gorczynski et al. (2017) also found no significant relationship between gender and attitudes towards seeking help. However, some studies suggest that female students have more positive attitudes towards seeking help than male students (Seyfi et al. 2013; Conceição, Rothes, and Gusmão 2022; Kantaş Yılmaz and Ünkür 2023). The lack of a significant difference in this study may be due to sample limitations, such as the gender distribution. The predominantly female sample (89%) aligns with studies indicating that female students often experience higher psychological vulnerability and lower academic life satisfaction (Nogueira, Sequeira, and Sampaio 2021). While gender did not play a significant role in this study, previous research suggests that it is a known predictor of positive mental health outcomes, which may have influenced the trends observed in this predominantly female group (Nogueira et al. 2022). Social concerns about masculinity and fear of stigmatisation may lead men to be less willing to seek help, whereas women may be more open to it. Societal expectations may discourage men from expressing vulnerability and seeking help, impacting their attitudes towards psychological support.

## 5 Conclusion

This study underscores the importance of understanding the demographic and educational factors that shape university students' mental health literacy and their attitudes towards seeking psychological help. The findings suggest that targeted interventions addressing these factors can play a critical role in fostering positive mental health behaviours. Enhancing mental health literacy among students is essential for early recognition and intervention, ultimately reducing barriers to help‐seeking and promoting psychological well‐being. Universities are uniquely positioned to implement educational initiatives, provide accessible psychological support services and engage multidisciplinary teams to support students' mental health needs.

Future research should explore broader populations and longitudinal impacts of mental health literacy programmes to develop evidence‐based strategies that further encourage help‐seeking behaviours and reduce stigma surrounding mental health. By addressing these challenges, educational institutions can significantly contribute to improving the mental health outcomes of young adults.

## 6 Relevance for Clinical Practice

Mental health literacy is a crucial determinant in increasing the rate of seeking psychological help when needed. Addressing the lack of knowledge concerning mental health is essential for the early diagnosis, prevention, protection and maintenance of mental health issues.

To enhance mental health literacy and attitudes towards seeking help among university students, the following are recommended:

1. Ensuring that psychological support services at universities are accessible and well‐publicised via websites, social media and physical postings.
2. Planning anti‐stigma and mental health promotion initiatives by university administrations and experts.
3. Engaging psychiatric nurses in collaborative multidisciplinary work with university academic and administrative units to educate students.
4. Include health and mental health literacy courses in the university curriculum.
5. May, which coincides with ‘Mental Health Awareness Month’ in the academic year, can be used to organise events, seminars and trainings at universities. These activities can motivate students to act regarding their mental health and direct them to appropriate resources.

Implementing these strategies can enhance mental health literacy and positively influence students' attitudes towards seeking psychological help, ultimately contributing to better mental health outcomes.

## Author Contributions

Study design: N.D.K. and G.K. Data collection: N.D.K. Data analysis: N.D.K. Study supervision: G.K. Manuscript writing: N.D.K. Critical revisions for important intellectual content: G.K. All authors read and approved the final manuscript.

## Ethics Statement

The study adhered to ethical guidelines throughout the research process. Permissions for using the MHLS and ATSPPHS‐SF were obtained from the original developers and the researchers who conducted the Turkish validation studies. Ethical approval was granted by the Haliç University Non‐Invasive Clinical Research Ethics Committee (Approval No: 2022/26), and institutional permission was obtained from the university (Approval No: 74). Participants were provided with detailed information about the study, including its purpose, procedures and confidentiality assurances, through an informed consent form. This form was included as a prerequisite for accessing the online survey. Data collection was conducted anonymously, and the responses were stored securely to protect participants' privacy. The study's ethical considerations ensured that participants' rights were prioritised, aligning with the principles of respect for persons, beneficence and justice.

## Conflicts of Interest

The authors declare no conflicts of interest.

## Supporting information


Data S1.

## Data Availability

The data that support the findings of this study are available on request from the corresponding author. The data are not publicly available due to privacy or ethical restrictions.
