# Supplementary material for: Determining the Mental Health Literacy Level of University Students and Examining Their Attitudes Towards Seeking Psychological Help
Source: Int J Ment Health Nurs. 2025 Jan 21;34(1):e13506. doi: 10.1111/inm.13506 (PMC11747956; doi:10.1111/inm.13506)
Supplement: Supplementary file 1 — Data S1. [file INM-34-0-s001.docx]

**Supplementary Table S1. Total Mean Scores of Participants from the Mental Health Literacy Scale (MHLS) and the Attitudes Towards Seeking Professional Psychological Help Scale (ATSPPHS)**

| **Scale (n=317)** | **Mean ± SD** | **Min-Max** |
| --- | --- | --- |
| **MHLS** – *Knowledge-oriented* | 7,94±2,08 | 0-10 |
| **MHLS** – *Belief-oriented* | 2,51±2,1 | 0-7 |
| **MHLS** – *Resource-oriented* | 2,69±1,13 | 0-4 |
| **MHLS - TOTAL** | 13,15±3,38 | 3-20 |
| **ATSPHS - TOTAL** | 18,38±3,77 | 6-29 |

*Abbreviation:* MHLS, Mental Health Literacy Scale

*Abbreviation:* ATSPPHS, Attention Towards Seeking Professional Psychological Help Scale

**Supplementary Table S2. Relationship between the age of the participants and the total and sub-dimensions of MHLS and ATSPPHS total score averages**

| **Scale (n=317)** | **Age** | |
| --- | --- | --- |
| **MHLS** – *Knowledge-oriented* | **r** | 0,202 |
|  | **p** | **0,000** |
| **MHLS** – *Belief-oriented* | **r** | 0,025 |
|  | **p** | 0,658 |
| **MHLS** – *Resource-oriented* | **r** | -0,019 |
|  | **p** | 0,739 |
| **MHLS - TOTAL** | **r** | 0,079 |
|  | **p** | 0,162 |
| **ATSPHS - TOTAL** | **r** | -0,018 |
|  | **p** | 0,750 |

r=Pearson Correlation

*Abbreviation:* MHLS, Mental Health Literacy Scale

*Abbreviation:* ATSPPHS, Attention Towards Seeking Professional Psychological Help Scale

**Supplementary Table S3. Difference between Participants' Characteristics and Information-Oriented MHLS Sub-Dimension Mean Scores**

| **Variables (n=317)** | | **MHLS – Knowledge-oriented** | | | |
| --- | --- | --- | --- | --- | --- |
|  |  | **Mean ± SD** | **Min-Max** | **Z** | **p** |
| **Gender** | Female | 7,94±2,09 | 0-10 | -0,109 | 0,913 |
|  | Male | 7,97±1,99 | 3-10 |  |  |
| **Employment status** | Working | 8,22±1,84 | 2-10 | -1,105 | 0,269 |
|  | Not working | 7,88±2,12 | 0-10 |  |  |
| **Took psychology course in undergraduate education** | Yes | 7,99±2,04 | 0-10 | -0,293 | 0,769 |
|  | No | 7,89±2,12 | 0-10 |  |  |
| **Previously received psychological support** | Yes | 7,75±2,29 | 0-10 | -0,643 | 0,520 |
|  | No | 8±2 | 0-10 |  |  |
| **Accuracy of psychology information on the internet** | True | 8,03±2,03 | 0-10 | -1,031 | 0,302 |
|  | False | 7,8±2,16 | 0-10 |  |  |
|  | | **Mean ± SD** | **Min-Max** | **Kwχ2** | **p** |
| **Department** | Nutrition and Dietetics | 7,59±2,63 | 0-10 | 0,045 | 0,978 |
|  | Midwifery | 7,7±2,6 | 0-10 |  |  |
|  | Physiotherapy and Rehabilitation | 8±1,48 | 6-10 |  |  |
|  | Nursing | 8,11±1,72 | 1-10 |  |  |
| **Grade** | 1st grade | 7,76±2,24 | 0-10 | 2,835 | 0,418 |
|  | 2nd grade | 8,18±1,98 | 0-10 |  |  |
|  | 3rd grade | 8,41±1,22 | 6-10 |  |  |
|  | 4th grade | 8,15±1,59 | 5-10 |  |  |
| **Living situation** | Living alone^a^ | 6±3,35 | 0-10 | 8,664 | **0,034**  **b>a** |
|  | Living in a dormitory^b^ | 8,49±1,44 | 4-10 |  |  |
|  | Living with family^c^ | 7,94±2,02 | 0-10 |  |  |
|  | Living with roommate/friends^d^ | 8±2,37 | 0-10 |  |  |
| **Mother’s education level** | Literate^a^ | 6,22±3,57 | 0-10 | 13,826 | **0,017**  **f>e>c>d** |
|  | Primary school^b^ | 8,08±1,69 | 1-10 |  |  |
|  | High school^c^ | 7,93±2,24 | 0-10 |  |  |
|  | Associate degree^d^ | 5,83±3,06 | 1-9 |  |  |
|  | Undergraduate^e^ | 8,74±1,01 | 6-10 |  |  |
|  | Postgraduate^f^ | 9,5±0,71 | 9-10 |  |  |
| **Father’s education level** | Literate | 8,67±1,03 | 8-10 | 6,702 | 0,244 |
|  | Primary school | 8,09±1,95 | 0-10 |  |  |
|  | High school | 7,78±2,37 | 0-10 |  |  |
|  | Associate degree | 8,29±1,27 | 6-10 |  |  |
|  | Undergraduate | 7,83±2,05 | 1-10 |  |  |
|  | Postgraduate | 6,33±2,35 | 3-10 |  |  |
| **Person providing psychological support** | Psychologist | 7,75±2,29 | 0-10 | 1,293 | 0,524 |
|  | Psychiatrist | 8±1,9 | 3-10 |  |  |
|  | Psychological counselor | 7,33±1,37 | 6-9 |  |  |
|  | Multiple experts | 7,47±3,29 | 0-10 |  |  |

Z=Mann Whitney U Kwχ2 = Kruskal Wallis

*Abbreviation:* MHLS, Mental Health Literacy Scale

**Supplementary Table S4. Difference between Participants' Characteristics and Belief-Oriented MHLS Sub-Dimension Mean Scores**

| **Variables (n=317)** | | **MHLS – Belief-oriented** | | | |
| --- | --- | --- | --- | --- | --- |
|  |  | **Mean ± SD** | **Min-Max** | **Z** | **p** |
| **Gender** | Female | 2,49±2,08 | 0-7 | -0,423 | 0,673 |
|  | Male | 2,71±2,31 | 0-7 |  |  |
| **Employment status** | Working | 2,69±2,26 | 0-7 | -0,587 | 0,557 |
|  | Not working | 2,47±2,07 | 0-7 |  |  |
| **Took psychology course in undergraduate education** | Yes | 2,52±2,16 | 0-7 | -0,096 | 0,924 |
|  | No | 2,51±2,05 | 0-7 |  |  |
| **Previously received psychological support** | Yes | 2,87±2,26 | 0-7 | -1,661 | 0,097 |
|  | No | 2,39±2,04 | 0-7 |  |  |
| **Accuracy of psychology information on the internet** | True | 2,56±2,14 | 0-7 | -0,510 | 0,610 |
|  | False | 2,43±2,04 | 0-7 |  |  |
|  | | **Mean ± SD** | **Min-Max** | **Kwχ2** | **p** |
| **Department** | Nutrition and Dietetics | 2,48±2,14 | 0-7 | 1,035 | 0,596 |
|  | Midwifery | 2,26±1,99 | 0-7 |  |  |
|  | Physiotherapy and Rehabilitation | 2,91±1,97 | 0-6 |  |  |
|  | Nursing | 2,56±2,13 | 0-7 |  |  |
| **Grade** | 1st grade | 2,44±2,03 | 0-7 | 2,955 | 0,399 |
|  | 2nd grade | 2,71±2,29 | 0-7 |  |  |
|  | 3rd grade | 3±2,2 | 0-7 |  |  |
|  | 4th grade | 2,04±1,97 | 0-7 |  |  |
| **Living situation** | Living alone^a^ | 2,14±1,83 | 0-6 | 2,011 | 0,570 |
|  | Living in a dormitory^b^ | 2,64±2,13 | 0-7 |  |  |
|  | Living with family^c^ | 2,54±2,11 | 0-7 |  |  |
|  | Living with roommate/friends^d^ | 2±2,31 | 0-7 |  |  |
| **Mother’s education level** | Literate^a^ | 2±1,91 | 0-6 | 2,856 | 0,722 |
|  | Primary school^b^ | 2,67±2,13 | 0-7 |  |  |
|  | High school^c^ | 2,38±2,09 | 0-7 |  |  |
|  | Associate degree^d^ | 2,5±2,59 | 0-6 |  |  |
|  | Undergraduate^e^ | 2,26±2,14 | 0-7 |  |  |
|  | Postgraduate^f^ | 2,5±0,71 | 2-3 |  |  |
| **Father’s education level** | Literate | 3±1,79 | 0-5 | 1,879 | 0,866 |
|  | Primary school | 2,52±2,1 | 0-7 |  |  |
|  | High school | 2,33±2,05 | 0-7 |  |  |
|  | Associate degree | 3±2,25 | 0-6 |  |  |
|  | Undergraduate | 2,63±2,39 | 0-7 |  |  |
|  | Postgraduate | 2,44±1,51 | 0-4 |  |  |
| **Person providing psychological support** | Psychologist | 2,75±2,52 | 0-7 | 1,987 | 0,370 |
|  | Psychiatrist | 3,03±2,1 | 0-7 |  |  |
|  | Psychological counselor | 1,67±1,51 | 0-4 |  |  |
|  | Multiple experts | 3,47±2,47 | 0-7 |  |  |

Z=Mann Whitney U Kwχ2 = Kruskal Wallis

*Abbreviation:* MHLS, Mental Health Literacy Scale

**Supplementary Table S5. Difference between Participants' Characteristics and Resource-Oriented MHLS Sub-Dimension Mean Scores**

| **Variables (n=317)** | | **MHLS – Resource-oriented** | | | |
| --- | --- | --- | --- | --- | --- |
|  |  | **Mean ± SD** | **Min-Max** | **Z** | **p** |
| **Gender** | Female | 2,68±1,13 | 0-4 | -0,517 | 0,605 |
|  | Male | 2,8±1,11 | 1-4 |  |  |
| **Employment status** | Working | 2,78±1,17 | 0-4 | -0,675 | 0,500 |
|  | Not working | 2,68±1,12 | 0-4 |  |  |
| **Took psychology course in undergraduate education** | Yes | 2,7±1,13 | 0-4 | -0,114 | 0,909 |
|  | No | 2,69±1,13 | 0-4 |  |  |
| **Previously received psychological support** | Yes | 2,96±1,11 | 0-4 | -2,593 | **0,010** |
|  | No | 2,61±1,12 | 0-4 |  |  |
| **Accuracy of psychology information on the internet** | True | 2,7±1,08 | 0-4 | -0,013 | 0,990 |
|  | False | 2,68±1,2 | 0-4 |  |  |
|  | | **Mean ± SD** | **Min-Max** | **Kwχ2** | **p** |
| **Department** | Nutrition and Dietetics | 2,59±1,05 | 0-4 | 3,884 | 0,143 |
|  | Midwifery | 2,65±1,16 | 0-4 |  |  |
|  | Physiotherapy and Rehabilitation | 1,91±1,22 | 0-4 |  |  |
|  | Nursing | 2,78±1,13 | 0-4 |  |  |
| **Grade** | 1st grade | 2,7±1,1 | 0-4 | 0,736 | 0,865 |
|  | 2nd grade | 2,73±1,07 | 0-4 |  |  |
|  | 3rd grade | 2,45±1,37 | 0-4 |  |  |
|  | 4th grade | 2,74±1,26 | 0-4 |  |  |
| **Living situation** | Living alone^a^ | 2,5±1,09 | 1-4 | 0,939 | 0,816 |
|  | Living in a dormitory^b^ | 2,68±1,2 | 0-4 |  |  |
|  | Living with family^c^ | 2,72±1,12 | 0-4 |  |  |
|  | Living with roommate/friends^d^ | 2,56±1,15 | 1-4 |  |  |
| **Mother’s education level** | Literate^a^ | 2,61±1,42 | 0-4 | 4,007 | 0,548 |
|  | Primary school^b^ | 2,68±1,13 | 0-4 |  |  |
|  | High school^c^ | 2,8±1,1 | 0-4 |  |  |
|  | Associate degree^d^ | 2,5±1,22 | 1-4 |  |  |
|  | Undergraduate^e^ | 2,57±0,99 | 1-4 |  |  |
|  | Postgraduate^f^ | 1,5±0,71 | 1-2 |  |  |
| **Father’s education level** | Literate | 3,67±0,82 | 2-4 | 6,443 | 0,265 |
|  | Primary school | 2,62±1,16 | 0-4 |  |  |
|  | High school | 2,72±1,1 | 1-4 |  |  |
|  | Associate degree | 3±1,11 | 1-4 |  |  |
|  | Undergraduate | 2,7±1,04 | 0-4 |  |  |
|  | Postgraduate | 2,67±1,22 | 1-4 |  |  |
| **Person providing psychological support** | Psychologist | 2,75±1,22 | 0-4 | 0,530 | 0,767 |
|  | Psychiatrist | 2,94±1,15 | 0-4 |  |  |
|  | Psychological counselor | 3,17±0,75 | 2-4 |  |  |
|  | Multiple experts | 3,07±1,03 | 1-4 |  |  |

Z=Mann Whitney U Kwχ2 = Kruskal Wallis

*Abbreviation:* MHLS, Mental Health Literacy Scale

**Supplementary Table S6. Difference between Participants' Characteristics and Total MHLS Mean Scores**

| **Variables (n=317)** | | **MHLS – TOTAL** | | | |
| --- | --- | --- | --- | --- | --- |
|  |  | **Mean ± SD** | **Min-Max** | **Z** | **p** |
| **Gender** | Female | 13,11±3,32 | 3-20 | -0,429 | 0,668 |
|  | Male | 13,43±3,82 | 4-20 |  |  |
| **Employment status** | Working | 13,42±3,52 | 4-20 | -0,196 | 0,845 |
|  | Not working | 13,09±3,35 | 3-20 |  |  |
| **Took psychology course in undergraduate education** | Yes | 13,25±3,44 | 3-20 | -0,604 | 0,546 |
|  | No | 13,04±3,32 | 3-20 |  |  |
| **Previously received psychological support** | Yes | 13,63±3,42 | 4-20 | -1,570 | 0,117 |
|  | No | 12,98±3,35 | 3-20 |  |  |
| **Accuracy of psychology information on the internet** | True | 13,37±3,23 | 3-20 | -1,453 | 0,146 |
|  | False | 12,79±3,58 | 4-20 |  |  |
|  | | **Mean ± SD** | **Min-Max** | **Kwχ2** | **p** |
| **Department** | Nutrition and Dietetics | 12,62±4,03 | 3-20 | 0,613 | 0,736 |
|  | Midwifery | 13,17±3,06 | 4-19 |  |  |
|  | Physiotherapy and Rehabilitation | 12,82±2,82 | 7-17 |  |  |
|  | Nursing | 13,34±3,24 | 3-20 |  |  |
| **Grade** | 1st grade | 12,99±3,43 | 3-20 | 1,635 | 0,651 |
|  | 2nd grade | 13,49±3,27 | 3-20 |  |  |
|  | 3rd grade | 13,59±2,58 | 9-20 |  |  |
|  | 4th grade | 12,85±3,89 | 4-20 |  |  |
| **Living situation** | Living alone^a^ | 12,07±4,41 | 4-18 | 3,567 | 0,312 |
|  | Living in a dormitory^b^ | 13,57±3,11 | 4-20 |  |  |
|  | Living with family^c^ | 13,22±3,33 | 3-20 |  |  |
|  | Living with roommate/friends^d^ | 11,75±3,7 | 4-17 |  |  |
| **Mother’s education level** | Literate^a^ | 12,22±3,86 | 4-17 | 2,555 | 0,768 |
|  | Primary school^b^ | 13,3±3,41 | 3-20 |  |  |
|  | High school^c^ | 13,23±3,04 | 4-20 |  |  |
|  | Associate degree^d^ | 10,67±5,82 | 3-19 |  |  |
|  | Undergraduate^e^ | 12,96±3,43 | 4-20 |  |  |
|  | Postgraduate^f^ | 13,5±0,71 | 13-14 |  |  |
| **Father’s education level** | Literate | 15,33±1,97 | 13-18 | 5,040 | 0,411 |
|  | Primary school | 13,21±3,24 | 4-20 |  |  |
|  | High school | 12,86±3,52 | 4-20 |  |  |
|  | Associate degree | 14,14±2,74 | 8-19 |  |  |
|  | Undergraduate | 12,93±3,85 | 3-20 |  |  |
|  | Postgraduate | 12,78±3,73 | 4-18 |  |  |
| **Person providing psychological support** | Psychologist | 13,38±3,54 | 4-20 | 0,603 | 0,740 |
|  | Psychiatrist | 13,67±3,23 | 4-20 |  |  |
|  | Psychological counselor | 13±1,1 | 12-14 |  |  |
|  | Multiple experts | 14,47±4,49 | 4-20 |  |  |

Z=Mann Whitney U Kwχ2 = Kruskal Wallis

*Abbreviation:* MHLS, Mental Health Literacy Scale

**Supplementary Table S7. Difference Between Participants' Characteristics and ATSPPHS Total Mean Scores**

| **Variables (n=317)** | | **ATSPPHS - TOTAL** | | | |
| --- | --- | --- | --- | --- | --- |
|  |  | **Mean ± SD** | **Min-Max** | **Z** | **p** |
| **Gender** | Female | 18,51±3,67 | 6-29 | -1,735 | 0,083 |
|  | Male | 17,34±4,38 | 7-27 |  |  |
| **Employment status** | Working | 18,55±3,44 | 12-27 | -0,329 | 0,742 |
|  | Not working | 18,34±3,84 | 6-29 |  |  |
| **Took psychology course in undergraduate education** | Yes | 18,6±3,78 | 7-29 | -0,541 | 0,588 |
|  | No | 18,16±3,75 | 6-29 |  |  |
| **Previously received psychological support** | Yes | 19,43±4,11 | 11-29 | -2,775 | **0,006** |
|  | No | 18,03±3,59 | 6-29 |  |  |
| **Accuracy of psychology information on the internet** | True | 18,26±3,93 | 6-29 | -0,439 | 0,660 |
|  | False | 18,57±3,5 | 7-29 |  |  |
|  | | **Mean ± SD** | **Min-Max** | **Kwχ2** | **p** |
| **Department** | Nutrition and Dietetics | 18,65±3,88 | 7-29 | 0,215 | 0,898 |
|  | Midwifery | 19,33±3,47 | 14-27 |  |  |
|  | Physiotherapy and Rehabilitation | 18,82±4,47 | 13-26 |  |  |
|  | Nursing | 18,04±3,73 | 6-29 |  |  |
| **Grade** | 1st grade | 18,26±3,67 | 6-29 | 0,815 | 0,846 |
|  | 2nd grade | 18,75±3,88 | 11-29 |  |  |
|  | 3rd grade | 18,36±3,74 | 13-26 |  |  |
|  | 4th grade | 18,15±4,27 | 7-25 |  |  |
| **Living situation** | Living alone^a^ | 18,71±4,7 | 11-27 | 1,998 | 0,573 |
|  | Living in a dormitory^b^ | 18,26±3,78 | 6-26 |  |  |
|  | Living with family^c^ | 18,45±3,7 | 7-29 |  |  |
|  | Living with roommate/friends^d^ | 17,38±4,03 | 12-27 |  |  |
| **Mother’s education level** | Literate^a^ | 16,94±3,3 | 12-26 | 7,673 | 0,175 |
|  | Primary school^b^ | 18,46±3,42 | 6-27 |  |  |
|  | High school^c^ | 18,73±3,93 | 7-29 |  |  |
|  | Associate degree^d^ | 17,5±3,15 | 13-22 |  |  |
|  | Undergraduate^e^ | 17,57±5,61 | 7-29 |  |  |
|  | Postgraduate^f^ | 19±0 | 19-19 |  |  |
| **Father’s education level** | Literate | 20,33±3,2 | 18-26 | 2,510 | 0,775 |
|  | Primary school | 18,47±3,83 | 6-29 |  |  |
|  | High school | 18,44±3,37 | 12-29 |  |  |
|  | Associate degree | 18,21±2,58 | 11-22 |  |  |
|  | Undergraduate | 17,82±4,8 | 7-29 |  |  |
|  | Postgraduate | 17,44±2,96 | 11-20 |  |  |
| **Person providing psychological support** | Psychologist | 19,04±4,41 | 12-27 | 0,591 | 0,744 |
|  | Psychiatrist | 19,53±4,06 | 11-29 |  |  |
|  | Psychological counselor | 18,83±1,17 | 17-20 |  |  |
|  | Multiple experts | 20,53±3,89 | 12-27 |  |  |

Z=Mann Whitney U Kwχ2 = Kruskal Wallis

*Abbreviation:* ATSPPHS, Attention Towards Seeking Professional Psychological Help Scale
